# Where to Draw Cerebrospinal Fluid from an External Ventricular Drain? Comparison of Cerebrospinal Fluid Parameters between Two Different Collection Sites

**DOI:** 10.3390/diagnostics13233543

**Published:** 2023-11-27

**Authors:** Farjad Khalaveh, Mario Mischkulnig, Vitalij Zeiser, Matthias G. Vossen, Andrea Reinprecht, Christian Dorfer, Karl Rössler, Johannes Herta

**Affiliations:** 1 Department of Neurosurgery, Medical University of Vienna, Waehringer Guertel 18-20, 1090 Vienna, Austria; farjad.khalaveh@meduniwien.ac.at (F.K.); mario.mischkulnig@meduniwien.ac.at (M.M.); vitalij.zeiser@meduniwien.ac.at (V.Z.); andrea.reinprecht@meduniwien.ac.at (A.R.); christian.dorfer@meduniwien.ac.at (C.D.); karl.roessler@meduniwien.ac.at (K.R.); 2 Department of Medicine I, Division of Infectious Diseases and Tropical Medicine, Medical University of Vienna, Waehringer Guertel 18-20, 1090 Vienna, Austria; matthias.vossen@meduniwien.ac.at

**Keywords:** external ventricular drain, CSF sampling, EVD-associated infection

## Abstract

Background: High cerebrospinal fluid (CSF) sampling frequency is considered a risk factor for external ventricular drain (EVD)-associated infections. To reduce manipulation at the proximal port and potentially minimize the risk of an infection, we aimed to analyze whether CSF parameters sampled from the far distal collection bag could provide reliable results compared to the proximal port. Methods: We included patients who were treated with an EVD at our neurosurgical intensive care unit (ICU) between June 2021 and September 2022. CSF sampling, including microbiological analysis, was performed simultaneously from the proximal port and the collection bag. Spearman’s correlation coefficients were calculated to assess the correlation of CSF cell count, protein, lactate and glucose between the two sample sites. Results: We analyzed 290 pairs of CSF samples in 77 patients. Ventriculitis was identified in 4/77 (5%) patients. In 3/4 patients, microbiological analysis showed the same bacterial species at both sample sites at the same time. Spearman’s correlation coefficient showed that CSF cell count (r = 0.762), lactate (r = 0.836) and protein (r = 0.724) had a high positive correlation between the two collection sites, while CSF glucose (r = 0.663) showed a moderate positive correlation. Conclusion: This study shows that biochemical CSF parameters can be reliably assessed from the EVD collection bag.

## 1. Introduction

External ventricular drain (EVD) placement is considered one of the most important life-saving treatment strategies in neurosurgical patients, particularly when dealing with patients facing acute hydrocephalus. The external ventricular drain provides continuous monitoring of intracranial pressure (ICP) and effective ICP reduction by a constant cerebrospinal fluid (CSF) release.

Despite its high diagnostic and treatment potential, EVDs are also associated with a high rate of infections [1,2,3,4,5]. Various risk factors have been extensively described in the literature [5,6,7]. A high CSF sampling frequency has been considered one of the most common risk factors in promoting EVD-associated infections [5,6,7]. To reduce infection rates, it has been proposed that CSF sampling should not be routinely performed and should be restricted to cases in which infections are suspected based on clinical parameters. However, predicting an EVD-associated infection in neurocritically ill patients is challenging since, in this patient cohort, laboratory and clinical parameters are often altered due to systemic infections or intraventricular hemorrhage [8]. Although microbiological analysis is considered to be the gold standard in identifying EVD-associated infections, the results are usually only available with considerable delay. Modern multiplex PCR systems have been developed to alleviate this drawback; however, they suffer from a limited spectrum of identifiable microorganisms [9]. Given the need for timely intervention and the limitations of current diagnostic approaches, sequential CSF parameter analysis remains of utmost importance. This approach can significantly enhance treatment strategies and consequently reduce infection-associated morbidity and duration of hospitalization.

Previous studies have explored CSF sampling from a more distal site, at the EVD drip chamber, aiming to reduce manipulation at the proximal port and potentially decrease the risk of EVD-associated infections while preserving data integrity [10,11]. These studies demonstrated that CSF samples from the EVD drip chamber yielded results comparable to those from the proximal port [10,11]. Nevertheless, to our knowledge, no previous study has analyzed the reliability of CSF parameters acquired from an even more distal site at the EVD collection bag. Hence, our objective was to evaluate the correlation between CSF parameters obtained from the far distal collection bag and those from the proximal port.

## 2. Materials and Methods

We retrospectively analyzed consecutive patients who were treated with an EVD at our neurosurgical intensive care unit (ICU) between June 2021 and September 2022. We included only patients who were 18 years or older with at least two concurrent CSF samples from the proximal port and the collection bag obtained on the same day. Patients who received an EVD due to meningitis or ventriculitis were excluded from our study.

We assessed the following variables: patient’s demographics, indication for EVD insertion (subarachnoid hemorrhage (SAH), tumor, trauma, intracerebral hemorrhage (ICH) or other), CSF cell count, protein, glucose, lactate and microbiological analysis of CSF from both collection sites and type of EVD-associated infection according to our previously published definition [7].

### 2.1. External Ventricular Drainage System

**Figure 1:**
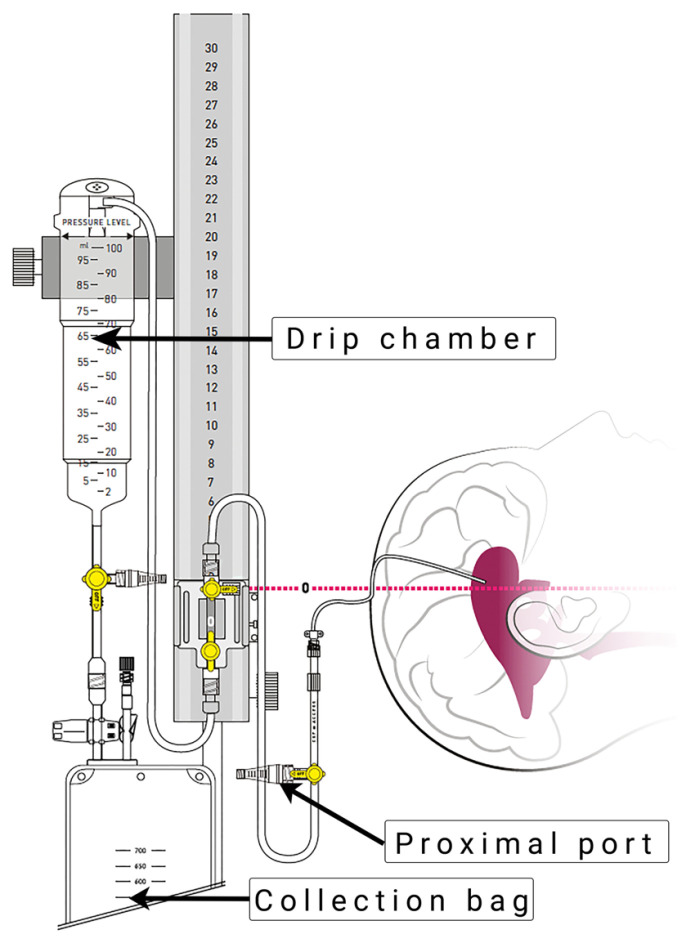
External ventricular drain system (VentrEX^®^ by Neuromedex^®^, Hamburg, Germany). Reprinted with permission from Neuromedex^®^. Copyright by Neuromedex^®^.

The EVD insertion technique and drainage system (Figure 1) were applied according to our routine hospital protocol, as previously described by Khalaveh et al. [7].

EVD insertion was performed by a neurosurgical consultant or resident in the operating room or in the ICU under sterile conditions. The procedures were performed under appropriate sedation and analgesia, with the patient in the supine position and the head of the bed elevated by 30–40 degrees. The area of insertions was shaved and prepared under sterile conditions. After identifying Kocher’s point, a 2 cm straight incision at the pupilar line was performed. A self-retaining retractor was placed, and a small burr hole was drilled. After coagulating and incising the dura, a regular non-antibiotic coated ventricular catheter (Straight Ventricular Catheter F8, Integra LifeSciences©, Princeton, NJ, USA) was passed toward the ipsilateral medial canthus and ipsilateral tragus into the frontal horn of the lateral ventricle. Subsequently, the catheter was tunneled under the galea and secured by multiple sutures.

### 2.2. CSF Sampling Protocol

According to our clinical protocol, CSF sampling was performed by the neurosurgeon assigned to the ICU from the 3-way stopcock at the proximal port twice a week. Additionally, since the EVD collection bag has to be changed every day, daily CSF samplings from the removed collection bag were performed as well. From each site, we withdrew 2 mL CSF to assess cell count, protein, glucose and lactate, and 4 mL for the microbiological analysis.

### 2.3. Definition of Infections

We used the definition of EVD-associated infections according to our previously published study (Table 1) [7]. According to our definition of EVD-associated infections, which is based on previously published studies and the guidelines of the Infectious Diseases Society of America (ISDA), patients were retrospectively classified into one of the five categories, depending on the microbiological results of testing CSF samples, which were drawn at the proximal port [4,8,9]. We further arranged our patients into the groups (1) “infection” or (2) “no-infection” (Table 1).

### 2.4. Statistical Analysis

Statistical analysis was performed using SPSS software version 28.0 (IBM, Armonk, NY, USA). Categorical data were presented as counts and percentages, and continuous parameters as median and range. To assess the correlation of CSF cell count, protein, lactate and glucose between two CSF samples, Spearman’s correlation coefficients were calculated. Interpretation of Spearman’s correlation coefficients was performed according to established ranges as previously described by Mukaka et al. [12]. A *p*-value < 0.05 was considered statistically significant for all performed tests.

## 3. Results

We analyzed 290 pairs of CSF samples in 77 patients treated with an EVD at a median age of 58 years (range: 18–80 years). There were 44 (57%) female patients. The reason for EVD placement was hydrocephalus due to SAH in 60 (78%) patients, intracerebral tumor in 8 (10%) patients and other diseases in 9 (12%) patients (including spontaneous intracerebral hemorrhage, ischemic stroke, trauma or autoimmune encephalitis).

### 3.1. CSF Sample Comparison between the Proximal Port and the Collection Bag

The median CSF cell count, glucose and lactate from the proximal port was 57 cells/µL (range: 0–17,720 cells/µL), 71 mg/dL (range: 11–113 mg/dL) and 2.8 mmol/L (range: 1.2–18.6 mmol/L), respectively. Quantitative protein values were available from 107 samples and showed a median value of 32 mg/dL (range: 0–1856 mg/dL).

The median CSF cell count, glucose, lactate and protein from the collection bag was 26 cells/µL (range: 0–2830 cells/µL), 69 mg/dL (range: 2–114 mg/dL), 2.9 mmol/L (range: 0.6–11.8 mmol/L) and 55.3 mg/dL (range: 4.3–797 mg/dL), respectively.

CSF cell count and glucose, acquired from the proximal site, showed median 1.84 (range: 0–910) and 1.01 (range: 0.39–6.2) times higher values compared to the distal site. In contrast, CSF lactate and protein showed median 0.93 (0.42–5.8) and 0.99 (range: 0–25.32) times lower values.

**Figure 2:**
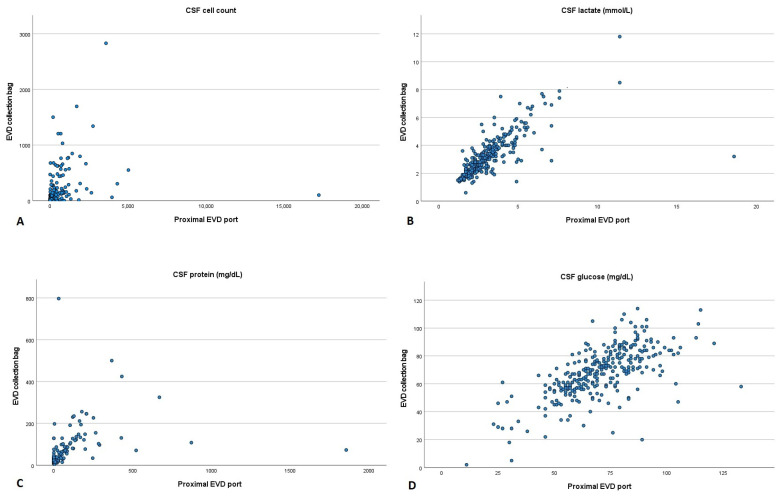
Scatter plot comparing cerebrospinal fluid parameters acquired from the proximal port and collection bag of an external ventricular drain. (**A**) Cell count, (**B**) lactate, (**C**) protein and (**D**) glucose. CSF: cerebrospinal fluid; EVD: external ventricular drain.

After calculating Spearman’s correlation coefficient, we could observe that CSF cell count (r = 0.762, *p* < 0.001), lactate (r = 0.836, *p* < 0.001) and protein (r = 0.724, *p* < 0.001) had a high positive correlation between the two collection sites. In contrast, CSF glucose (r = 0.663, *p* < 0.001) showed a moderate positive correlation (Figure 2).

### 3.2. Comparison of Microbiological Results of CSF between the Two Collection Sites

To analyze whether microbiological analysis could be reliably assessed from the collection bag as well, we further compared the microbiological results from the two collection sites. In regard to infection status, which was based on the proximal results of the microbiological analysis, 73/77 (95%) patients had no infections, with 9/77 (12%) classified as contamination and 4/77 (5%) patients as ventriculitis.

The results of the positive microbiological analysis from the proximal EVD port showed 19 bacterial species in 17 samples, with *Staphylococcus epidermidis* (7/19; 37%) being the most frequent species. *Staphylococcus* spp. was identified in all nine contamination cases. In the ventriculitis group, *Candida albicans* and *Escherichia coli* 4 MRGN were identified separately in three consecutive microbiological analyses in one patient, respectively. In the other two cases, the microbiological analysis revealed *Serratia marcescens* and *Klebsiella pneumoniae*.

In one patient with ventriculitis (*Candida albicans* and *Staphylococcus epidermidis*) and in one with contamination (*Staphylococcus hominis* and *Staphylococcus epidermidis*), two species were identified in the same microbiological analysis.

The microbiological analysis of the CSF from the collection bag showed *Staphylococcus epidermidis* (10/16; 63%) as the most frequent species. In three out of four cases of ventriculitis, microbiological analysis identified the same species as from the proximal EVD port on the same day. Moreover, in one case, *Serratia marcescens* was identified 2 days prior to the positive result of the proximal sample. We have summarized all microbiological results from the proximal EVD port and the collection bag and compared them to each other in Table 2.

## 4. Discussion

This study aimed to investigate whether CSF parameters acquired from the EVD collection bag would show comparable results to CSF parameters collected from the proximal port of the EVD system. We showed that CSF cell count, lactate and protein levels had a high positive correlation between the two sample sites. Therefore, these parameters could be reliably assessed from CSF drawn at the more distal EVD collection bag to potentially decrease EVD-associated infections by reducing manipulation at the proximal site of the EVD system [5,6,7].

There are various guidelines available on how to effectively utilize CSF parameters in the diagnosis of specific diseases affecting the central nervous system, including conditions like bacterial infections [13]. These recommendations may not be universally applicable, especially when dealing with a specific subset of patients—the neurocritically ill individuals who require treatment through an EVD. This becomes particularly pertinent in cases following intraventricular hemorrhage (IVH) or subarachnoid hemorrhage (SAH). In these patients, CSF parameters may be altered, which reduces their diagnostic reliability. Therefore, the Infectious Diseases Society of America (IDSA) has stressed that the primary basis for diagnosing EVD-associated infections should rest on the outcomes derived from CSF cultures [9]. This approach has been integrated into our department’s protocols. In addition to implementing these guidelines, we intended to analyze whether microbiological analyses from the CSF collection bag could yield results of comparable diagnostic value to those obtained from the proximal port of the EVD. Our data revealed that in three out of four (75%) ventriculitis cases, the microbiological findings from both collection sites (distal and proximal) concurred, and, moreover, in one case, the microbiological analysis from the distal site showed a positive result two days prior to the proximal sample. Furthermore, the rate of contamination species at the distal site was comparable to that at the proximal site, with 10 cases compared to 9, which minimizes the concerns about an elevated risk of contamination at the distal sample site. We believe that assessing CSF cultures from the collection bag could have the potential to be as reliable as the proximal assessment while concurrently mitigating the risk of inadvertently introducing bacterial species into the ventricles through the proximal EVD port. Thus, assessment from the collection bag is not limited by sample frequency as it would be from the proximal port. However, it is imperative to acknowledge that irrespective of the sampling site, the microbiological results may take several days to reach their conclusive stage, and these results may be influenced by any ongoing empirical antibiotic therapy. To improve the diagnosis of bacterial infections, contemporary techniques, such as *polymerase chain reaction* (PCR) or metagenomic next-generation sequencing, have been introduced into the clinical setting [14,15,16]. Yet, these techniques are either limited in their spectrum of detectable pathogens or are expensive and are, therefore, not routinely used in daily clinical practice.

Since a one-time CSF parameter analysis in neurosurgical patients is not specific enough to detect EVD-associated infections, clinicians at the neurosurgical ICU need to rely on the sequential analysis of CSF parameters to prevent a delay in antibiotic treatment and to guide treatment decisions [17,18]. Our study showed that CSF parameter analysis from the collection bag would generate comparable results to those from the proximal site with the potential to prevent a loss of information and guide treatment decisions. Similar results have been found in previous studies comparing CSF parameters from the EVD drip chamber to the proximal collection site [10,11]. While Wong et al. showed that CSF protein and glucose correlated between the two collection sites, Kinast et al. could demonstrate that CSF lactate, protein and glucose could be reliably determined from the distal site [10,11].

In contrast to these studies, our study showed only a moderate positive correlation of glucose values between the two collection sites. According to Deisenhammer et al., CSF glucose degrades during storage, which could explain our results [13]. Another explanation could be a dilution effect of constantly altered glucose values since sampling from the collection bag has been performed after CSF accumulation over 24 h. However, there are no data in regard to the appropriate method of CSF storage, with recommendations being inconsistent compared to published data [19]. While it has been emphasized by the Guidelines of the European Federation of Neurological Societies (EFNS) that glucose sample processing should be performed immediately after acquisition, others have shown that glucose concentrations remain stable over 2.5 h at room temperature or even over 24 h at 4 °C [13,20,21]. Nevertheless, studies that analyzed CSF glucose acquired from the EVD drip chamber have not specified the exact duration of CSF storage [10,11].

Compared to glucose, the stability of CSF lactate and protein concentrations seems to be less affected by storage time. While the data on the stability of these CSF parameters are scarce, it has been shown in previous studies that a time delay of 2.5 h in sample processing did not influence the analytical results [21]. Moreover, it has been shown that both remain stable for up to 3 days at room temperature [20,22,23]. Our results confirm previously published data and show that CSF lactate and protein samples can be reliably assessed from the EVD collection bag.

In regard to the CSF cell count, it appears that the in vitro biochemical process has been analyzed in more detail. Historical studies have shown that due to the hypotonic state of CSF, leukocytes lyse if they are exposed for an extended time period [24]. This cell degradation process has been confirmed by recent studies, in which it has been shown that even a short delay in CSF analysis leads to a statistically significant decrease in CSF cell count [21,25]. Since CSF collection at the drip chamber or collection bag occurs over many hours, one would assume that sampling CSF from these distal sites would result in lower cell count values compared to samples acquired from the proximal site. This has been observed by Kinast et al. and was confirmed by the results of the present study [10]. According to our results, the CSF cell count from the proximal site was median 1.84 times higher than the values from the distal site. This apparent cell count degradation process has not been previously analyzed in neurosurgical patients after SAH or IVH. Nevertheless, our study showed a high positive correlation of CSF cell count between the two sample sites, with similar results being described by Wong et al. [11]. Moreover, in studies with non-neurosurgical patients, it has been shown that a 5 h time delay of sample processing had no significant influence on the cell count analysis if a serum-containing medium was added to the CSF [25]. Thus, it could be hypothesized that in patients with SAH or IVH, the degradation process occurs only to a certain degree since, theoretically, these patients have a higher CSF osmolarity.

In summary, our data show that CSF parameters can be reliably assessed from the EVD collection bag. CSF sampling by this approach has the potential to reduce manipulation of the proximal site of the EVD system and, thus, theoretically reduce EVD-associated infections to a certain degree. However, since this is the first study analyzing the reliability of this CSF sampling approach, more studies are needed to validate our results.

## 5. Strength and Limitations

To our knowledge, this is the first study analyzing the analytic value of CSF parameters from the EVD collection bag. Moreover, the continuously applied standard CSF sampling protocol with a high amount of CSF sample pairs is the major strength of our study.

While we could show a moderate to high positive correlation of CSF values between the two collection sites, it must be pointed out that laboratory reference values are validated for CSF specimens acquired from the proximal site. Thus, future studies are needed to assess appropriate reference values for CSF parameters acquired from far distal collection sites.

Although we included neurosurgical patients only, our cohort still comprised a heterogeneous group of cases with a history of SAH in 78% of our patients. Moreover, due to our low infection rate (4/77 patients, 5%), we did not perform a comparison analysis of the microbiological results between the two collection sites. Thus, the comparison of the microbiological results should be interpreted with caution since this part of the study was purely descriptive.

## 6. Conclusions

Clinicians still depend on the sequential analysis of CSF parameters to detect EVD-associated infections due to (1) the lack of specific parameters and (2) the delayed availability of microbiological results. Moreover, microbiological analysis may be influenced by empiric antibiotic treatment. With the aim of reducing manipulation at the proximal port of the EVD system, we analyzed whether CSF parameters could be reliably detected from the far distal collection bag. Our results showed a high correlation of CSF cell count, lactate and protein between the two collection sites. Therefore, we are convinced that an increased CSF sampling frequency from the far distal collection bag is possible, with the potential to prevent a loss of information and minimize the risk of EVD-associated infections. This approach could guide the treating clinician to improve treatment strategies.

However, in cases of abnormal CSF values, CSF parameter analysis should always be performed from the proximal site to verify the analytical results. More importantly, future studies should focus on the development of newer techniques to expedite the determination of EVD-associated infections.

## Figures and Tables

**Table 1:** Classification of EVD-associated infection.

**No-infection**	**Negative microbiological results**	Negative CSF cultures
	**Contamination**	An isolated positive CSF culture with coagulase-negative Staphylococci or *Cutibacterium acnes* with a time to positivity >24 h
**Infection**	**Colonization**	Multiple positive CSF cultures with coagulase-negative Staphylococci or Cutibacteria with a time to positivity within 15 h
	**Ventriculitis**	Colonization in combination with an increased C-reactive protein at the time of CSF sampling Single or multiple positive CSF cultures containing *Enterobacter cloacae*, *Staphylococcus aureus*, *Serratia* spp., *Streptococcus* spp. and/or *Enterococcus faecalis*

Reprinted from Ref. [7]. CSF: cerebrospinal fluid.

**Table 2:** Comparison of the microbiological results of CSF between the proximal EVD port and the collection bag.

		Proximal EVD Port							
		No Infection	Ventriculitis				Contamination		
			*Serratia marcescens*	*Klebsiella pneumoniae*	*Candida albicans*	*Escherichia coli* 4 MRGN	*Staphylococcus capitis*	*Staphylococcus epidermidis*	*Staphylococcus hominis*
**EVD collection bag**	Negative results	268	1	1	0	0	1	1	2
	*Serratia marcescens*	1	0	0	0	0	0	0	0
	*Klebsiella pneumoniae*	0	0	0	0	0	0	0	0
	*Candida albicans*	0	0	0	2	0	0	0	0
	*Escherichia coli* 4 MRGN	0	0	0	0	3	0	0	0
	*Staphylococcus capitis*	0	0	0	0	0	0	0	0
	Staphylococcus epidermidis *	4	0	0	1	0	0	6	1
	*Staphylococcus hominis*	0	0	0	0	0	0	0	0

A total of 19 bacterial species were identified in the microbiological analysis of the proximal port. The results were compared to the bacterial species detected from the collection bag. Corresponding matches are visually highlighted in grey. * *Staphylococcus epidermidis* was identified in 10 samples, while in two patients, the analysis from the proximal port identified two bacterial species within the same microbiological assessment. EVD: external ventricular drain; MRGN: multidrug-resistant Gram-negative.

## Data Availability

The data presented in this study are available upon reasonable request from the corresponding author.
